# Sustained HIV viral suppression among men who have sex with men in the Miami-Dade County Ryan White Program: the effect of demographic, psychosocial, provider and neighborhood factors

**DOI:** 10.1186/s12889-020-8442-1

**Published:** 2020-03-13

**Authors:** Diana M. Sheehan, Rahel Dawit, Semiu O. Gbadamosi, Kristopher P. Fennie, Tan Li, Merhawi Gebrezgi, Petra Brock, Robert A. Ladner, Mary Jo Trepka

**Affiliations:** 1 Department of Epidemiology, Robert Stempel College of Public Health and Social Work, Florida International University, Miami, FL USA; 2 Center for Substance Use and HIV/AIDS Research on Latinos in the United States (C-SALUD), Florida International University, Miami, FL USA; 3 Research Centers in Minority Institutions (RCMI), Florida International University, 11200 SW 8th St, AHC 5, Room 479, Miami, FL 33199 USA; 4 Division of Natural Sciences, New College of Florida, 5800 Bayshore Rd, Sarasota, FL 34243 USA; 5 Department of Biostatistics, Robert Stempel College of Public Health and Social Work, Florida International University, 11200 SW 8th St, Miami, FL 33199 USA; 6 Behavioral Science Research Corporation, 2121 Ponce de Leon Blvd, Suite 240, Coral Gables, FL 33134 USA

**Keywords:** Human immunodeficiency virus, Viral suppression, Sustained viral suppression, Durable viral suppression, Men who have sex with men

## Abstract

**Background:**

HIV viral suppression is associated with health benefits for people living with HIV and a decreased risk of HIV transmission to others. The objective was to identify demographic, psychosocial, provider and neighborhood factors associated with sustained viral suppression among gay, bisexual, and other men who have sex with men.

**Methods:**

Data from adult men who have sex with men (MSM) enrolled in the Miami-Dade County Ryan White Program (RWP) before 2017 were used. Sustained viral suppression was defined as having an HIV viral load < 200 copies/ml in all viral load tests in 2017. Three-level (individual, medical case management site, and neighborhood) cross-classified mixed-effect models were used to estimate adjusted odds ratios (aOR) and 95% confidence intervals (CI) for sustained viral suppression.

**Results:**

Of 3386 MSM, 90.8% were racial/ethnic minorities, and 84.4% achieved sustained viral suppression. The odds of achieving sustained viral suppression was lower for 18–24 and 25–34 year-old MSM compared with 35–49 year-old MSM, and for non-Latino Black MSM compared with White MSM. Those not enrolled in the Affordable Care Act, and those with current AIDS symptoms and a history of AIDS had lower odds of achieving sustained viral suppression. Psychosocial factors significantly associated with lower odds of sustained viral suppression included drug/alcohol use, mental health symptoms, homelessness, and transportation to appointment needs. Individuals with an HIV physician who serves a larger volume of RWP clients had greater odds of sustained viral suppression. Neighborhood factors were not associated with sustained viral suppression.

**Conclusion:**

Despite access to treatment, age and racial disparities in sustained viral suppression exist among MSM living with HIV. Addressing substance use, mental health, and social services’ needs may improve the ability of MSM to sustain viral suppression long-term. Furthermore, physician characteristics may be associated with HIV outcomes and should be explored further.

## Background

Suppression of the human immunodeficiency virus (HIV) (< 200 copies/mL) among people living with HIV (PLHIV) is associated with immune reconstruction [1] and a decreased risk of acquired immune deficiency syndrome (AIDS)-defining conditions [2] and death [3]. In addition, viral suppression prevents the transmission of HIV to others [4]. A recent analysis of National HIV Surveillance System and National HIV Behavioral Surveillance data showed a rate of 0 per 100 person-years of HIV transmission for individuals on antiretroviral therapy (ART) with suppressed viral loads, but a rate of 6.1 per 100 person-years for individuals in care who were not virally suppressed [4].

Despite these benefits, only 61.2% of men who have sex with men (MSM) with HIV in the United States showed evidence of viral suppression in 2014 [5]. Black MSM showed particularly low rates of viral suppression (52.2%) compared with non-Latino White MSM (61.2%) [5]. Young MSM also show concerning rates of viral suppression. Further, the intersection of race/ethnicity and young age put minority MSM at risk, with Black MSM aged 20–24 years showing viral suppression rates as low as 45.3% compared with 60.7% of 20–24 year old White MSM [5].

A significant limitation of previous research on viral suppression has been the use of a single viral load test, typically the last of a given year, to determine an individual’s suppression status. A study of adult PLHIV in care found that a single test measure overestimates sustained viral suppression (all tests in a year < 200 copies/mL) by approximately 16% [6]. Moreover, sustained viral suppression was lower among MSM compared with heterosexual individuals, with 22% of MSM having both suppressed and non-suppressed laboratory tests in the 12-month study period [6]. Non-Latino Blacks and young individuals were also more likely to have fluctuating viral suppression results compared with non-Latino Whites and older PLHIV, respectively [6]. Similarly, a 2018 Centers for Disease Control and Prevention (CDC) study found that MSM with HIV who were in care but who did not achieve sustained viral suppression spent approximately 46.8% of days in the year with viral loads > 1500 copies/mL [7]. Black MSM spent 53.4% days of the year with viral loads > 1500 copies/mL compared with 37.8% of the year for Whites [7].

These findings from previous studies have strong implications for transmission risk. However, little is known about the factors that affect PLHIV’s ability to sustain viral suppression long-term, including MSM. Thus, the objective of this study was to identify demographic, psychosocial, provider and neighborhood factors associated with sustained viral suppression among gay, bisexual, and other men who have sex with men.

## Methods

### Study population and study design

Data from adult (18 years or older) gay, bisexual and other men who have sex with men enrolled in the Miami-Dade County Ryan White Program (RWP) Part A/Minority AIDS Initiative (MAI) [8] any time before January 2017 were used to conduct a cross-sectional study. Enrollment in the RWP was defined as having at least one medical case management encounter or peer education support network service during 2017. RWP clients are required to have a comprehensive health assessment every 6 months. Thus, individuals without a client health assessment in 2016 or 2017 were considered not enrolled and excluded. Additionally, those referred for ancillary services to the RWP by a non-RWP HIV provider, and those whose client file was closed because of movement to another county/state, financial ineligibility, or incarceration were excluded (11.9%). Male RWP clients were classified as gay, bisexual and other men who have sex with men (herein after referred to as “MSM”) if they reported any MSM behavior.

### Outcome

HIV viral load data were obtained from viral load laboratory test results. Sustained viral suppression was defined as having a HIV viral load < 200 copies/ml in all viral load tests in 2017. A viral load of < 200 copies/ml was chosen to stay consistent with the Centers for Disease Control and Prevention (CDC) definition for an undetectable viral load and to be able to compare to national studies [9]. If any test in 2017 was ≥200 copies/ml (not suppressed), they were considered not to have achieved sustained viral suppression in 2017. If an individual had only 1 viral load test in 2017 (or more than 1 test but the tests were less than 3 months apart) and the test showed viral suppression, we looked to the last viral load test in 2016 in an effort to assess consistent viral suppression on at least 2 tests [7]. Individuals with only 1 suppressed viral load test in 2017 and no viral load tests in 2016, or with no viral load tests in 2017 were excluded (*n* = 216; 6.0%).

### Predictors

Demographic data were obtained from the client intake assessment conducted at time of entry into the RWP. Demographic variables considered included age, race/ethnicity, birth region, and preferred language. Race was categorized into non-Latino Black, Latino, and non-Latino White. Due to small numbers of Haitians (*n* = 46), those reported as Haitian were categorized based on data about their race and Latino ethnicity (non-Latino Black = 43, Latino = 2, White = 1). Also because of small numbers, those with race other than Black or White (Asian = 21, Native American/Alaskan Native = 5, Native Hawaiian/Pacific Islander = 2), and those with unknown Latino ethnicity (*n* = 3) were excluded.

Psychosocial data were obtained from the first comprehensive health assessment conducted in 2017 and pertain to the clients’ responses at that time [8]. We chose the psychosocial variables based on the Behavioral Model for Vulnerable Populations [10] and what was available from the comprehensive health assessment, and grouped the variables into need and vulnerable/enabling characteristics. Need characteristics included AIDS diagnosis, and current HIV-related symptoms. Vulnerable/enabling characteristics included 30 variables related to mental health, substance and alcohol use, HIV status disclosure, employment/disability status, income, household structure, access to transportation, social services need, and social support (see Additional File 1 for details). Individuals with missing data on psychosocial indicators were categorized into the lower risk groups (e.g. no alcohol use). We developed indices of vulnerable/enabling factors to reduce collinearity by conducting reliability analysis and exploratory factors analysis, followed by confirmatory factor analysis. In reliability analysis, we removed 13 vulnerable/enabling variables which increased the Cronbach’s alpha from 0.33 to 0.62. Factor analysis using Varimax rotation yielded 5 indices: substance use, mental health, housing and transportation needs, unemployment, and food insecurity and low social support. Factor loadings are provided in Additional File 1; one variable was deleted due to a factor loading < 0.4 (experience of domestic violence). See Table 1 for variables included in each index.

**Table 1:** Characteristics of men who have sex with men by sustained viral load suppression, 2017 (*n* = 3386)

	Total	Sustained viral load suppression		*P*-value^a^
		No, *n* (%)	Yes, *n* (%)	
Demographic characteristics				
Age group (years)				< 0.0001
18–24	191	51 (26.7)	140 (73.3)	
25–34	817	163 (20.0)	654 (80.0)	
35–49	1292	183 (14.2)	1109 (85.8)	
50+	1086	132 (12.2)	954 (87.8)	
Race				< 0.0001
Black	446	141 (31.6)	305 (68.4)	
Hispanic	2630	348 (13.2)	2282 (86.8)	
White	310	40 (12.9)	270 (87.1)	
Birth region				< 0.0001
United States	847	194 (22.9)	653 (77.1)	
Mexico/Central America	342	58 (17.0)	284 (83.0)	
Caribbean	1348	186 (13.8)	1162 (86.2)	
South America	765	80 (10.5)	685 (89.5)	
Other	84	11 (13.1)	73 (86.9)	
Mainland US Born				< 0.0001
Yes (excludes Puerto Rico, US Virgin Island and other US territories)	847	194 (22.9)	653 (77.1)	
No	2539	335 (13.2)	2204 (86.8)	
Preferred language				< 0.0001
English	1239	255 (20.6)	984 (79.4)	
Spanish	2081	261 (12.5)	1820 (87.5)	
Other	66	13 (19.7)	53 (80.3)	
Health care environment characteristics				
Number of Ryan White clients that client’s physician cares for				< 0.0001
1–9	72	6 (8.3)	66 (91.7)	
10–29	97	16 (16.5)	81 (83.5)	
30–99	537	113 (21.0)	424 (79.0)	
100–199	910	138 (15.2)	772 (84.8)	
200+	1585	212 (13.4)	1373 (86.6)	
Unknown	185	44 (26.8)	141 (76.2)	
Enrolled in the Affordable Care Act health insurance exchange				< 0.0001
Yes	643	53 (8.2)	590 (91.8)	
No	2743	476 (17.4)	2267 (82.6)	
HIV/AIDS-related health status				
AIDS symptoms (current)				< 0.0001
Yes	36	19 (52.8)	17 (47.2)	
No	3350	510 (15.2)	2840 (84.8)	
Diagnosis of AIDS (history)				< 0.0001
Yes	1021	208 (20.4)	813 (79.6)	
No	2365	321 (13.6)	2044 (86.4)	
Factors in substance use index				
Mean (SD)	−0.02 (0.96)	0.34 (1.44)	−0.09 (0.82)	
Median (IQR)	−0.33 (0)	−0.33 (0.74)	− 0.33 (0)	< 0.0001
Alcohol use				< 0.0001
Used alcohol in the last 12 months	418	110 (26.3)	308 (73.7)	
Did not use alcohol in last 12 months	2968	419 (14.1)	2549 (85.9)	
Drug use				< 0.0001
Used drugs in the last 12 months	235	85 (36.2)	150 (63.8)	
Did not use drugs in the last 12 months	3151	444 (14.1)	2707 (85.9)	
Drug use resulted in problems with daily activities/legal issue/hazardous situation				< 0.0001
Yes	56	29 (51.8)	27 (48.2)	
No	3330	500 (15.0)	2830 (85.0)	
Drug use affected adherence				< 0.0001
Yes	172	57 (33.1)	115 (66.9)	
No	3214	472 (14.7)	2742 (85.3)	
Factors in mental health index				
Mean (SD)	−0.01 (0.99)	0.33 (1.26)	−0.08 (0.91)	
Median (IQR)	−0.49 (0)	− 0.49 (1.24)	− 0.49 (0)	< 0.0001
Feeling depressed or anxious				< 0.0001
Yes	488	130 (26.6)	358 (73.4)	
No	2898	399 (13.8)	2499 (86.2)	
Having difficulty sleeping				< 0.0001
Yes	359	99 (27.6)	260 (72.4)	
No	3027	430 (14.2)	2597 (85.8)	
Receives or needs mental health services				< 0.0001
Yes	486	132 (27.2)	354 (72.8)	
No	2900	397 (13.7)	2503 (86.3)	
Factors in housing and transportation index				
Mean (SD)	−0.01 (0.99)	0.38 (1.49)	−0.08 (0.84)	
Median (IQR)	−0.32 (0)	− 0.32 (0)	−0.32 (0)	< 0.0001
Homeless				< 0.0001
Yes	133	51 (38.4)	82 (61.7)	
No	3253	478 (14.7)	2775 (85.3)	
Client needs help with transportation to appointments				< 0.0001
Yes	230	77 (33.5)	153 (66.5)	
No	3156	452 (14.3)	2704 (85.7)	
Factors in household structure index				
Mean (SD)	0.01 (1.03)	−0.06 (0.68)	0.02 (1.08)	
Median (IQR)	−0.21 (0)	− 0.21 (0)	−0.21 (0)	0.0805
Household size				0.2042^b^
One	3071	492 (16.1)	2579 (84.0)	
Two	264	30 (11.4)	234 (88.6)	
Three	38	6 (15.8)	32 (84.2)	
Four or more	13	1 (7.7)	12 (92.3)	
Number of minors in household				0.3747^b^
None	3315	519 (15.7)	2796 (84.3)	
One	54	9 (16.7)	45 (83.3)	
Two	13	0 (0.0)	13 (100.0)	
Three or more	4	1 (25.0)	3 (75.0)	
Lives with minor only				0.5987^b^
Yes	6	0 (0.0)	6 (100.0)	
No	3380	529 (15.7)	2851 (84.4)	
Factors in unemployment index				
Mean (SD)	−0.02 (0.99)	0.19 (1.01)	−0.06 (0.98)	
Median (IQR)	−0.56 (1.28)	− 0.56 (1.28)	−0.56 (1.28)	< 0.0001
Disability that prevents working				0.5154
Yes	246	42 (17.1)	204 (82.9)	
No	3140	487 (15.5)	2653 (84.5)	
Unemployed				<.0001
Yes	994	237 (23.8)	757 (76.2)	
No	2392	292 (12.2)	2100 (87.8)	
Factors in food insecurity and low social support index				
Mean (SD)	−0.008 (0.99)	0.12 (1.24)	−0.03 (0.93)	
Median (IQR)	−0.41 (0)	−0.41 (0)	− 0.41 (0)	0.0578
Client does not have social support system				0.1721
Yes	626	109 (17.4)	517 (82.6)	
No	2760	420 (15.2)	2340 (84.8)	
Client not getting food he needs				0.0009
Yes	53	17 (32.1)	36 (67.9)	
No	3333	512 (15.4)	2821 (84.6)	
Neighborhood deprivation index				
Mean (SD)	0.34 (0.86)	0.51 (0.90)	0.31 (0.85)	
Median (IQR)	0.15 (1.25)	0.34 (1.56)	0.15 (1.12)	< 0.0001
Neighborhood residential instability and crime index				
Mean (SD)	0.52 (1.07)	0.60 (1.01)	0.50 (1.08)	
Median (IQR)	0.41 (1.71)	0.65 (1.61)	0.41 (1.74)	0.0315

*AIDS* acquired immune deficiency syndrome, *US* United States, *SD* standard deviation, *IQR* interquartile range

^a^ Chi-square test p-value; ^b^ Fisher’s test p-value

To assess provider factors, we calculated the number of RWP clients by HIV physician and assigned this number to all clients who are primarily served by that physician. Additionally, we considered whether the client was enrolled in the Affordable Care Act (ACA) health insurance exchange. Neighborhood data were obtained from the 2013–2017 American Community Survey (ACS) by zip code tabulation area (ZCTA) and merged using client’s residential ZIP code [11]. The number of homicides for each ZIP code in Miami-Dade County was obtained from SimplyAnalytics [12]. A total of 25 neighborhood variables were considered (see Additional file 2). Variables were related to 5 categories: socioeconomic status, racial/ethnic composition, language and US nativity, residential instability, and violent crime. We developed indices as described above which yielded 2 indices: neighborhood deprivation, and residential instability and crime.

### Analytical plan

All analyses were conducted in SAS Version 9.4. Chi-squared (or Fisher’s exact test when applicable) for categorical variables and Wilcoxon signed-ranked test for continuous variables were used to compare sustained viral suppression by demographic, psychosocial, provider, and neighborhood characteristics in bivariate analyses. Three-level (individual, medical case management site, and neighborhood) cross-classified mixed-effects models were used to estimate adjusted odds ratios (aOR) and 95% confidence intervals (CI) for sustained viral suppression in multivariate logistic regression analyses using a residual pseudo-likelihood estimation technique in the PROC GLIMMIX procedure. Individuals were allowed to cluster by medical case management site and neighborhood, but these were mutually exclusive groups; the intraclass correlation coefficient (ICC) was calculated for each possible combination. We first fitted a cross-classified model that included demographic, provider and need factors, and indices of psychosocial and neighborhood factors (each index was tested as the combined standardized score of the variables in the index). Variable selection was guided by the Behavioral Model for Vulnerable Populations [10] and the literature. Variables in Table 2 were entered all at once into the model. Model fit was assessed by ensuring the ratio of the generalized chi-square statistic and the degrees of freedom was close to 1. For each psychosocial or neighborhood index that was significant in the first model, we fitted separate cross-classified models that included demographic, need and provider factors, and all other indices with each variable in the index of interest one by one. We examined the influence of outliers by inspecting plots and estimates of the Pearson residuals, the deviance residuals, and the DFBETAS. We assessed multicollinearity by examining the Pearson correlation coefficients between all variables in the model and Type II Tolerance values. We conducted sensitivity analyses to assess the influence of missing data by comparing results to models excluding individuals with missing data, and by setting the missing values at all possible levels of a given variable.

**Table 2:** Factors associated with sustained viral suppression among men who have sex with men

Variable	AOR	95% CI	
Age group, years			
18–24	**0.511**	**0.341**	**0.766**
25–34	**0.676**	**0.523**	**0.874**
35–49	ref		
50+	1.260	0.973	1.630
Race			
Black	**0.444**	**0.288**	**0.685**
Hispanic	0.784	0.502	1.226
White	ref		
Not born in mainland US	0.987	0.700	1.392
Preferred language			
Spanish	1.016	0.740	1.396
Other	0.800	0.391	1.635
English	ref		
Number of Ryan White clients that client’s physician cares for			
1–9	2.304	0.947	5.605
10–29	1.459	0.782	2.722
30–99	ref		
100–199	**1.453**	**1.062**	**1.989**
200+	**1.459**	**1.101**	**1.935**
Unknown	0.850	0.546	1.325
Client not enrolled in the ACA	**0.657**	**0.478**	**0.903**
AIDS symptoms (current)	**0.258**	**0.123**	**0.540**
Diagnosis of AIDS	**0.580**	**0.467**	**0.721**
Substance use index	**0.816**	**0.747**	**0.891**
Mental health index	**0.814**	**0.743**	**0.891**
Housing and transportation index	**0.817**	**0.750**	**0.890**
Household structure index	**1.169**	**1.009**	**1.353**
Unemployment index	0.918	0.830	1.015
Food insecurity and social support index	0.950	0.868	1.039
Neighborhood deprivation index	0.952	0.843	1.075
Neighborhood residential instability and crime index	0.977	0.886	1.078

*AOR* adjusted odds ratio, *CI* confidence intervals, *ACA* Affordable Care Act

Bolded values show significant values

## Results

Of 3386 MSM enrolled in the Miami-Dade County RWP before 2017, 90.8% were racial/ethnic minorities, with 77.7% being Latino and 75.0% being foreign-born. In 2017, 84.4% of MSM achieved sustained viral suppression. The results of the bivariate analyses are presented in Table 1.

In the multivariate analyses the odds of sustained viral suppression were lower for younger MSM (18–24 [aOR 0.51, 95% CI 0.34–0.77] and 25–34 [aOR 0.68, 95% CI 0.52–0.87] compared with 35–49 year-olds) and for Black MSM compared with White MSM (aOR 0.44, 95% CI 0.29–0.69) (Table 2). Individuals not enrolled in the ACA (aOR 0.66, 95% CI 0.48–0.90), currently reporting AIDS symptoms (aOR 0.26, 95% CI 0.12–0.54), and with a history of AIDS diagnosis (aOR 0.58, 95% CI 0.47–0.72) had lower odds of sustained viral suppression. After controlling for demographic, need, provider, and neighborhood factors, four psychosocial indices were associated with lower odds of sustained viral suppression: substance use (aOR 0.82, 95% CI 0.75–0.89), mental health (aOR 0.81, 95% CI 0.74–0.89), housing and transportation (aOR 0.82, 95% CI 0.75–0.89), and household structure (aOR 1.17, 95% CI 1.01–1.35). Individuals with an HIV physician who served a larger volume of RWP clients had greater odds of sustained viral suppression (100–199 [aOR 1.45, 95% CI 1.06–1.99] and 200+ [aOR 1.46, 95% CI 1.10–1.94] compared with 30–99 clients). Neighborhood indices were also not associated with sustained viral suppression.

**Fig. 1:**
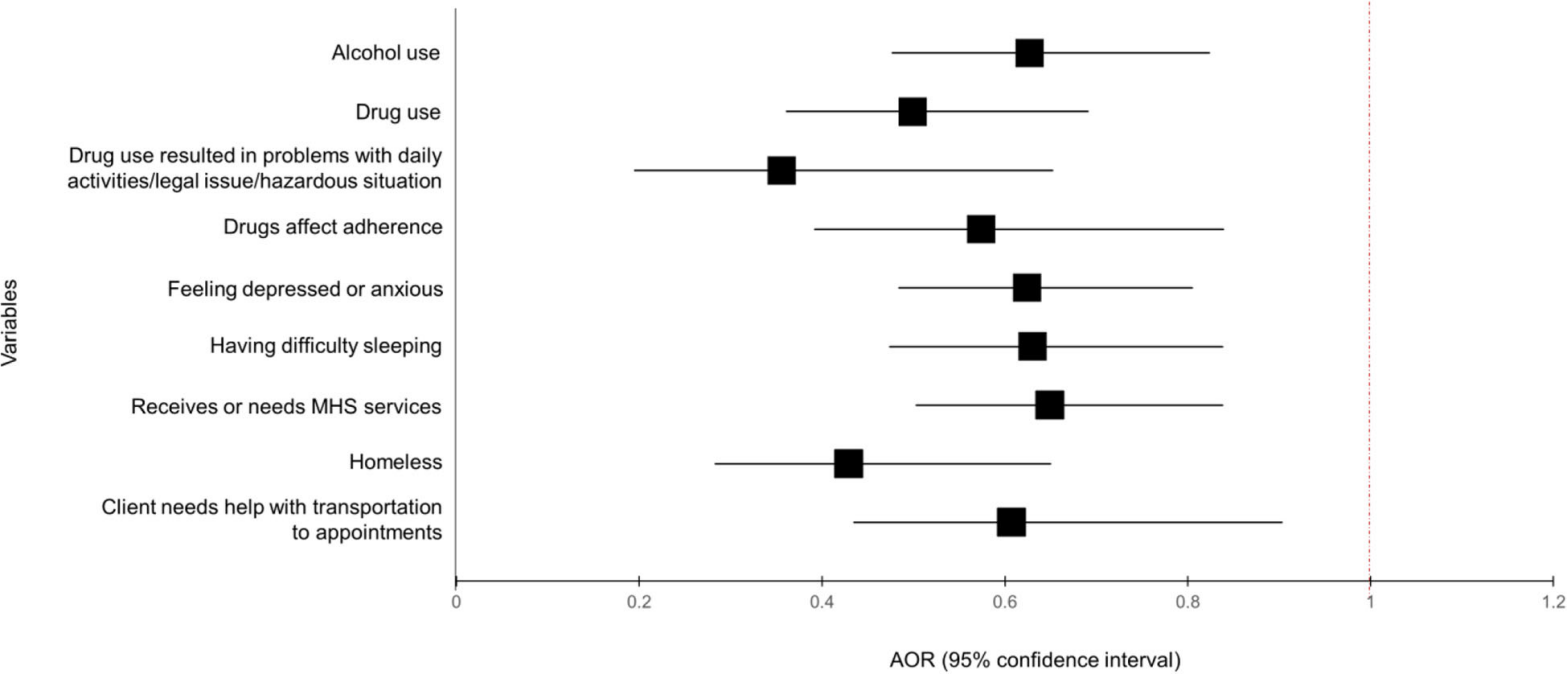
Factors associated with sustained viral suppression among men who have sex with men. Footnote: AIDS, acquired immune deficiency syndrome; MHS, mental health services; AOR, adjusted odds ratio. AORs adjusted for age, race, US born status, preferred language, number of Ryan White clients HIV physician cares for, client enrolled in Affordable Care Act, psychosocial indices (except index of interest), and neighborhood indices

When disaggregating the indices that were significant in the first model, we found most individual variables were strongly associated with lower odds of sustained viral suppression (Fig. 1). This included alcohol use (0.63, 0.48–0.82), drug use (0.50, 0.36–0.69), drug use resulted in problems (0.36, 0.20–0.65), drug use prevented ART adherence (0.57, 0.39–0.84), anxiety or depression (0.62, 0.48–0.81), difficulty sleeping (0.63, 0.47–0.84), needing or receiving mental health services (0.65, 0.50–0.84), homelessness (0.43, 0.28–0.65), and needing transportation help to appointments (0.61, 0.44–0.85).

Regarding the psychometrics of the indices, we assessed convergent validity by calculating the correlation coefficient (CC) between each psychosocial and neighborhood index and sustained viral suppression and all indices were negatively and significantly associated with sustained viral suppression (*p*-value < 0.001) as expected, except for the household structure index and residential instability indices which did not reach significance. Regarding fitting the cross-classified model, the ratio of the generalized chi-square statistic and degrees of freedom was 1.01 indicating the variability of the data was properly modeled and that there was no residual overdispersion. The ICCs were as follows: same ZIP code and same medical case management site 0.067; same ZIP code and different medical case management site 0.031; different ZIP code and same medical case management site 0.036. The analysis of outliers identified one outlier in the housing structure index; removing the outlier did not change the results. Multicollinearity was not observed; correlations between variables were < 0.8 and Type II Tolerance values were > 0.1. There were no missing data on demographic factors and only 1–4 (0.1%) missing values for mental health variables. Sensitivity analysis categorizing the missing data as missing, “no” (main analysis), and “yes” did not change the findings or statistical significance of the results.

## Discussion

Among a sexual minority and predominantly racial/ethnic minority population of MSM living with HIV, nearly 85% had evidence of sustained viral suppression. Our analyses resulted in four important findings. First, young MSM were significantly less likely to achieve sustained viral suppression. Second, racial disparities existed in sustained viral suppression, with Black MSM significantly less likely to achieve sustained viral suppression when compared with White MSM. Third, MSM experiencing drug/alcohol use, mental health symptoms including difficulty sleeping, homelessness and reporting a need for transportation help to appointments were less likely to achieve sustained viral suppression. Finally, clients with providers serving a larger volume of RWP clients were more likely to achieve sustained viral suppression. Notably, neighborhood factors were not associated with sustained viral suppression.

Our rate of sustained viral suppression among MSM (84.4%) was higher than that reported in previous national studies [7, 13–16]. This may be due to a significant upward trend in sustained viral suppression overtime nationwide [13, 17] and among PLHIV enrolled in the Ryan White Program [18] since previous studies used data from 2009 to 2016 and our study used 2017 data. Furthermore, previous studies have focused on the general population of PLHIV. Studies have reported that sustained viral suppression is higher among men compared with women [19] and among MSM compared with all other HIV risk groups [7].

Our finding that young MSM had lower odds of sustained viral suppression is consistent with national studies of the general population of PLHIV [7, 9, 13–15]. A study of 33 United States jurisdictions with complete reporting of viral load tests found that in 2014, 13–24 year old PLHIV were more likely to have unsuppressed viral loads at both their first and last test of the year and were more likely to have worsening viral load status (first test suppressed and last test unsuppressed) compared with all other age groups [15]. Further, a study of New York City HIV surveillance data found that younger PLHIV were more likely to have at least 2 consecutive viral loads ≥100,000 copies/mL [20]. It is worth noting that a study by Mandgasar et al. [18] suggested that age gaps in viral suppression among Ryan White Program clients decreased 2010–2016, thus disparities may be declining. However, this finding was not specific to MSM.

We identified racial disparities in sustained viral suppression with Black MSM having lower odds of sustained viral suppression when compared with White MSM. Our finding in Florida is consistent with findings at the national level for the general population of PLHIV [7, 13–15]. Although sustained viral suppression among Blacks has increased significantly in recent years [13], our findings suggest that Blacks continue to be disproportionately affected by challenges in sustaining viral suppression. Nevertheless, a study of Ryan White Program clients found significant declines in the difference between Blacks and Whites who were virally suppressed from 2010 (13.0 percentage point difference) to 2016 (8.1 percentage point difference) [18]. Worth noting, the percentage of undiagnosed infections is disproportionately higher among some groups including young MSM compared with older MSM and Black MSM compared with White MSM [21]. Given rates of undiagnosed infection, our study may underestimate disparities in sustained viral suppression among MSM. In a post hoc analysis, we tested the interaction between age and race/ethnicity but found it to be nonsignificant (*p*-value 0.3084).

After disaggregating the indices of psychosocial factors, we found that alcohol and drug use, reporting that drugs resulted in problems, and reporting that drugs prevented ART adherence were associated with decreased odds of sustained viral suppression. We were unable to identify other studies that examined the effect of these drug and alcohol-use related variables on sustained viral suppression with the exception of a study that found that reporting injection drug use (IDU) as an HIV risk factor was associated with decreased odds of sustained viral suppression compared with MSM behavior [17]. Of note, our study included 47 MSM that also reported IDU as an HIV risk exposure. Our findings are particularly important among MSM, as the prevalence of drug and alcohol use among this population is high [22], and drug use has been associated with poor ART adherence [23]. A recent study suggested that anticipated substance use stigma is associated with ART adherence among drug users, even after controlling for severity of drug and alcohol use [24]. MSM with HIV may experience multiple stigmas related to their sexual orientation, their HIV status and substance using behavior. Thus, addressing the compounded stigmas in this population may be one mechanism to target to increase sustained viral suppression.

Additionally, we found that feelings of anxiety or depression, difficulty sleeping, receiving or needing mental health services, as well as reporting homelessness were associated with decreased odds of sustained viral suppression. While studies have examined mental health [25] and homelessness [23, 26] as it relates to viral suppression at one given time, none have looked at these factors in relation to consistent viral suppression which requires long-term ART adherence. A study of homeless PLHIV receiving ART found that 31% of study participants discontinued ART. Among those who discontinued ART, only 51% were adherent to ART and 9% had viral loads < 400 copies/mL [23]. Further, among clients of a Ryan White Part-A funded Care Coordination Program (CCP), viral suppression was higher among those who were homeless at baseline but who obtained stable housing post-baseline compared with those who remained homeless [26]. It is important to discuss the interaction of these psychosocial factors (substance use, mental health, and homelessness) because MSM who report drug use or binge drinking are more likely to report having unstable living environments and having a severe mental health disorder [27]. These factors appear to be harder to address as a previous study showed smaller decreases in viral suppression among Ryan White Program clients who experience unstable or temporary housing compared with those with stable housing over a 6-year period [18]. Our study also suggests other social service’s needs, such as reporting a need for transportation help to appointments, may also affect sustained viral suppression consistent with barriers to linkage to HIV care identified in a qualitative study of US clinics [28].

Clients with providers serving a larger volume of RWP clients were more likely to achieve sustained viral suppression in our study. Our study was only able to measure the volume of RWP clients a physician sees, not the volume of all HIV patients. Our findings are consistent with several other studies that found HIV physician volume associated with HIV care and treatment outcomes [29–31]. Our findings may reflect provider experience with caring for PLHIV, or characteristics of the clinics in which they practice. Clinics with providers that serve a large volume of RWP clients likely also have medical case managers that are well versed in RWP requirements and who also have substantial expertise in serving a racial/ethnic diverse and low socioeconomic status population with significant social services needs and psychosocial barriers. Thus, more research is needed to better understand the role of physician characteristics in HIV outcomes. Additionally, other provider factors, which we were unable to measure, may also be important. A longitudinal cohort study in Baltimore among PLHIV with a history of injection drug use found that only having the same HIV provider > 90% of the time was associated with decreased odds of virologic failure [32].

Being enrolled in the ACA was associated with sustained viral suppression in our study. The effect of the ACA may be due to differences in income as clients must have an income of at least 100% of the federal poverty level (FPL) to be eligible for ACA. In post hoc analyses, we found that sustained viral suppression among those below 100% of FPL was 74.4% compared with 88.7% for those with incomes ≥100% of FPL (*p*-value < 0.0001). Of note, similar to our study, the two national studies by Crepaz et al. [15] and Bradley et al. [13] that found disparities in sustained viral suppression across age and race/ethnicity included only people receiving care. Bradley et al. [13] further controlled for ART prescription but disparities remained, suggesting factors other than access to care and treatment are important in sustained viral suppression such as the psychosocial factors identified in this study.

Finally, neighborhood factors were not associated with sustained viral suppression in our study. Our finding is inconsistent with one study of New York City surveillance data which suggested that while neighborhood poverty was not associated with achieving viral suppression, it was associated with lower likelihood of maintaining viral suppression after diagnosis [33]. However, the literature on the effect of neighborhoods on viral suppression is mixed with some studies showing an association between residing in areas of high deprivation and poor viral suppression [34, 35], and others showing no association [36, 37]. It is possible that neighborhood units smaller than the ZIP code or other neighborhood characteristics not measured in this study, particularly perceptions of one’s neighborhood, may be important. For example, a study found an association between perceived neighborhood disorder and ART non-adherence [38].

Our study has several limitations. The RWP serves PLHIV who are uninsured; thus, they are not representative of all MSM living with HIV. A second limitation relates to our definition of sustained viral suppression. While we are able to confidently say that those with only 1 viral load test that was ≥200 copies/ml did not achieve sustained viral suppression, we had to exclude those with only 1 viral load of < 200 copies/ml. We decided to exclude these clients because with only 1 viral load test result, we were unable to determine whether they were consistently suppressed. We compared demographic variables for those with missing vs. no missing sustained viral suppression data and found that those with missing data were more likely to be 34 years old or younger (*p*-value <.0001), black (*p*-value 0.0027), US born (*p*-value <.0001), English speaking (*p*-value <.0001), and less likely to be enrolled in the Affordable Care Act, all factors associated with not achieving sustained viral suppression. Finally, data were collected by numerous medical case managers for the purposes of service delivery. Thus, psychosocial information, particularly data about substance/alcohol use and mental health, were not collected using validated questionnaires, clinical evaluation tools, or procedures, and included only dichotomous yes/no response options.

## Conclusions

Despite these limitations, to our knowledge, our study is the first to examine characteristics at various levels (i.e. individual, provider, and neighborhood), together with psychosocial factors, that may be playing a role in the ability of MSM with HIV to sustain their viral loads suppressed over time. Our analyses showed that, despite access to treatment, age and racial disparities in sustained viral suppression exist among MSM living with HIV. Addressing substance use, mental health, and social services’ needs may improve the ability of MSM to sustain viral suppression long-term. Further, physician characteristics may be associated with HIV outcomes and should be explored further.

## Supplementary information

**Additional file 1.** Variables considered for health need and psychosocial indices

**Additional file 2.** Variables considered for neighborhood indices

## Data Availability

The data that support the findings of this study are available from Miami-Dade County but restrictions apply to the availability of these data, and so the data are not publicly available. Permission can be requested by contacting the Miami-Dade County Ryan White Program.

## Abbreviations

ACA: Affordable Care Act
ACS: American Community Survey
AIDS: Acquired Immune Deficiency Syndrome
aOR: Adjusted Odds Ratio
ART: Antiretroviral Therapy
CC: Correlation Coefficient
CDC: Centers for Disease Control and Prevention
CI: Confidence Intervals
FPL: Federal Poverty Level
HIV: Human Immunodeficiency Virus
MAI: Minority AIDS Initiative
MSM: Men Who Have Sex with Men
PLHIV: People living with HIV
RWP: Ryan White Program
ZCTA: Zip Code Tabulation Area
