# Supplementary material for: Sustained HIV viral suppression among men who have sex with men in the Miami-Dade County Ryan White Program: the effect of demographic, psychosocial, provider and neighborhood factors
Source: BMC Public Health. 2020 Mar 13;20:326. doi: 10.1186/s12889-020-8442-1 (PMC7069036; doi:10.1186/s12889-020-8442-1)
Supplement: Supplementary file 1 — Additional file 1. Variables considered for health need and psychosocial indices [file 12889_2020_8442_MOESM1_ESM.docx]

| **Additional File 1. Variables considered for psychosocial indices and rotated factor loadings** | | |
| --- | --- | --- |
| Factor | Description of variable | Rotated Factor Loading |
| Substance use | Are you currently using alcohol? | 0.61849 |
|  | Are you currently using drugs? (intravenous or other illicit drugs) | 0.90743 |
|  | Has drug use resulted in any problem in daily activity or legal issue or hazardous situation? | 0.59939 |
|  | When using drugs do you remain adherent to your ARV regimen? | 0.87294 |
| Mental health | Have you been feeling depressed or anxious? | 0.85332 |
|  | Have you been having difficulty sleeping? | 0.82785 |
|  | Needs referral to mental health counseling or currently receiving mental health services | 0.64231 |
| Unemployment | Do you have a disability that prevents you from working? | 0.85507 |
|  | Are you working? (reverse coded) | 0.79999 |
| Housing and transportation | Homeless | 0.67809 |
|  | Do you need assistance with transportation to your appointments? | 0.73553 |
| Household structure | Household size | 0.57531 |
|  | Number of minors in house | 0.73165 |
|  | Lives with minor ONLY | 0.72398 |
| Food insecurity and low social support | Are you getting the food you need? (reverse coded) | 0.65694 |
|  | Do you have a social support system you can depend on? | 0.78404 |
| Variables removed to improve reliability of the indices based on Cronbach’s alpha | Ever experienced domestic violence? |  |
|  | Does your partner know your HIV status? (reverse coded) |  |
|  | HIV status disclosure to the people with whom they live with |  |
|  | Does the client’s employer know about their HIV status? (reverse coded) |  |
|  | Do you have work-related barriers to attending appointments? |  |
|  | Getting Housing Opportunities for Persons with AIDS program services? |  |
|  | Is the client receiving Housing and Urban Development benefits? |  |
|  | Lives alone |  |
|  | Lives with spouse or partner/together |  |
|  | Percent of FPL on assessment date |  |
|  | Currently having difficulty getting medications |  |
|  | Does the client have access to transportation for health care/dental/social services appointment? |  |
|  | Do you attend a support group? |  |
|  | Have you had more than one sexual partner in the last 12 months? |  |
