# Supplementary material for: Sustained HIV viral suppression among men who have sex with men in the Miami-Dade County Ryan White Program: the effect of demographic, psychosocial, provider and neighborhood factors
Source: BMC Public Health. 2020 Mar 13;20:326. doi: 10.1186/s12889-020-8442-1 (PMC7069036; doi:10.1186/s12889-020-8442-1)
Supplement: Supplementary file 2 — Additional file 2. Variables considered for neighborhood indices [file 12889_2020_8442_MOESM2_ESM.docx]

| Additional file 2. Variables considered for neighborhood indices | |  |
| --- | --- | --- |
| Indices | Description of variable | Factor loadings |
| Neighborhood deprivation | Receiving public assistance | 0.50967 |
|  | Households without access to a car | 0.73343 |
|  | Households with ≥ 1 person per room | 0.81099 |
|  | Population living below the 2017 poverty line ($12,488 for one person) | 0.92761 |
|  | Owner-occupied homes worth ≤ $300,000 | 0.80435 |
|  | Households with annual income < $15,000 | 0.87884 |
|  | Households with annual income ≤ $150,000 | 0.84413 |
|  | Households with annual income <$10,000 divided by the percent of households with annual income ≥$50,000 | 0.90389 |
|  | Aged ≥ 25 with less than a 12th grade education | 0.85472 |
|  | Aged ≥ 25 with a graduate professional degree | 0.77590 |
|  | Households living in rented housing | 0.67080 |
|  | Aged ≥ 16 who were unemployed | 0.74075 |
|  | Aged ≥ 16 employed in high working-class occupation (ACS occupation group: ‘‘managerial, business, science, and arts occupations’’) | 0.87390 |
|  | Non-Hispanic Black | 0.51664 |
|  | Speaks English well for all populations | 0.45081 |
| Residential instability and crime | Moved within same county | 0.69500 |
|  | Number of homicides by zip code | 0.69573 |
| Removed Variables | Median household income in 2013 |  |
|  | Latino |  |
|  | US Born |  |
|  | Non-Hispanic White |  |
|  | Living in same house 1 year ago |  |
|  | Moved from different county within same state |  |
|  | Moved from different state |  |
|  | Moved from abroad |  |
